# THE IMPACT OF THE LIFE TRIAL ON CARDIOVASCULAR HEALTH: RACIAL DISPARITIES IN LIFESTYLE FACTOR IMPROVEMENT

**DOI:** 10.1093/geroni/igae098.1368

**Published:** 2024-12-31

**Authors:** Donya Nemati, Deepishka Pemmasani, Vaishali Lavangu, Navin Kaushal

**Affiliations:** The Ohio State University, Columbus, Ohio, United States; Indiana University Indianapolis, Indianapolis, Indiana, United States; Indiana University Indianapolis, Indianapolis, Indiana, United States; Indiana University Indianapolis, Indianapolis, Indiana, United States

## Abstract

Introduction. Cardiovascular disease is the leading cause of death among older adults. The American Heart Association promotes the lifestyle 8 (LE8) for cardiovascular disease prevention which includes healthy levels of exercise, sleep, smoking, BMI, blood pressure, nutrition (sodium), triglycerides and glucose levels. Exercise alone can improve most of these factors. The purpose of this study was to test if an exercise program can improve LE8 and identify racial (Black vs. White) differences. Methods. The LIFE Study is a randomized controlled trial that randomized 1,600 older adults (age 65+) to an intervention or control group (health-education program). The intervention included one-hour on-site exercise program twice per week with additional prescription of home-based exercise. LE8 measures included: exercise (accelerometers), sleep (Pittsburg-Sleep-Quality-Index), smoking (self-report). Blood samples measured glucose, triglycerides, and sodium, and on-site assessments measured blood pressure and BMI. Data was collected every six months for two years. STATA 18.0 was used to conduct multilevel modeling for each LE8 outcome. Results. A series of multilevel models revealed significant TimeXGroupXRace interaction effects for greater exercise(β=.12, p=.006), better sleep(β=.20, p=.04), lower BMI(β=.58, p<.001) and triglycerides(β=.38, p<.001) only among White participants in the intervention arm at the fifth timepoint. Sodium(β=.13, p=.04), glucose(β=.09, p=.04), and blood pressure(β=.20, p=.04) were higher in Black participants with no time or group interaction. Smoking was nonsignificant Conclusion. The LIFE trial demonstrated improvement in some LE8 factors, and several racial health disparities. There is a need to understand biopsychosocial determinants that contribute to racial disparities to ensure that all participants benefit equally.

